# Silicon Vacancies Diamond/Silk/PVA Hierarchical Physical Unclonable Functions for Multi‐Level Encryption

**DOI:** 10.1002/advs.202308337

**Published:** 2024-04-04

**Authors:** Fuhang Jiao, Chaonan Lin, Lin Dong, Xin Mao, Yi Wu, Fuying Dong, Zhenfeng Zhang, Junlu Sun, Shunfang Li, Xun Yang, Kaikai Liu, Lijun Wang, Chong‐Xin Shan

**Affiliations:** ^1^ Henan Key Laboratory of Diamond Optoelectronic Materials and Devices Key Laboratory of Materials Physics Ministry of Education School of Physics and Microelectronics Zhengzhou University Zhengzhou 450052 P. R. China; ^2^ MOE Key Laboratory of Fundamental Physical Quantities Measurement Hubei Key Laboratory of Gravitation and Quantum Physics PGMF School of Physics Huazhong University of Science and Technology Wuhan 430074 P. R. China

**Keywords:** anticounterfeit, diamond, phase separation, physical unclonable function, silicon vacancy

## Abstract

Physical unclonable functions (PUFs) have emerged as a promising encryption technology, utilizing intrinsic physical identifiers that offer enhanced security and tamper resistance. Multi‐level PUFs boost system complexity, thereby improving system reliability and fault tolerance. However, crosstalk‐free multi‐level PUFs remain a persistent challenge. In this study, a hierarchical PUF system that harnesses the spontaneous phase separation of silk fibroin /PVA blend and the random distribution of silicon‐vacancy diamonds within the blend is presented. The thermodynamic instability of phase separation and inherent unpredictability of diamond dispersion gives rise to intricate random patterns at two distinct scales, enabling time‐efficient hierarchical authentication for cryptographic keys. These patterns are complementary yet independent, inherently resistant to replication and damage thus affording robust security and reliability to the proposed system. Furthermore, customized authentication algorithms are constructed: visual PUFs authentication utilizes neural network combined structural similarity index measure, while spectral PUFs authentication employs Hamming distance and cross‐correlation bit operation. This hierarchical PUF system attains a high recognition rate without interscale crosstalk. Additionally, the coding capacity is exponentially enhanced using M‐ary encoding to reinforce multi‐level encryption. Hierarchical PUFs hold significant potential for immediate application, offering unprecedented data protection and cryptographic key authentication capabilities.

## Graphical abstract

A hierarchical PUF system is obtained by exploiting the spontaneous phase separation of the silk fibroin/PVA blend and the random distribution of silicon‐vacancy (SiV) diamonds within the blend. The visual PUF based on random phase separation contours and the spectral PUFs based on the stable luminescence of SiV diamond can be used individually for authentication or in combination, as needed, thus affording robust security and reliability to the hierarchical PUF system.

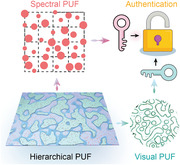

## 1 Introduction

Counterfeiting poses a pervasive global challenge, disrupting market stability, causing substantial economic losses, and even threatening human health and information security.^[^ ^1^, ^2^ ^]^ In response, numerous anticounterfeiting techniques have been invented, aiming to render the reproduction of genuine products more difficult for counterfeiters.^[^ ^3^ ^]^ Techniques like metasurface encryption,^[^ ^4^, ^5^ ^]^ holographic color printing,^[^ ^6^ ^]^ and fluorescent security inks^[^ ^7^, ^8^ ^]^ have gained traction and are considered promising candidates with significant application potential in the field of anticounterfeiting. Nevertheless, most of these techniques rely on deterministic production processes, which can be vulnerable to duplication over time due to advances in precision processing technology and the constant pursuit of new replication methods by counterfeiters. To address these security vulnerabilities, PUFs offer a promising alternative solution, leveraging stochastic physical features to generate unique and nonreproducible identifiers.^[^ ^9^, ^10^ ^]^

A diverse array of random physical features, including multicolor luminescence,^[^ ^11^, ^12^, ^13^ ^]^ Raman scattering,^[^ ^14^, ^15^ ^]^ spatial diffractions,^[^ ^16^ ^]^ structural color,^[^ ^17^ ^]^ supersaturated solution crystallization,^[^ ^18^ ^]^ phase separation,^[^ ^19^ ^]^ and symmetry breaking^[^ ^20^ ^]^ have been employed to implement PUFs. These systems utilize nonalgorithmic one‐way functions, generating distinct and unpredictable outputs (responses) in response to specific external stimuli (challenges), effectively acting as fingerprints.^[^ ^21^ ^]^ While some progress has been made, many existing PUFs are single‐mode challenge responses, which may require costly equipment or complex synthesis. Furthermore, the growing significance of enhanced encryption for secure authentication and data transmission highlights the need for multi‐level anticounterfeiting systems with heightened security and reliability. These systems play a crucial role in safeguarding personal intellectual property and sensitive information from counterfeiting threats. Therefore, the development of multi‐level PUF‐based anticounterfeiting technologies holds great promise in addressing these challenges effectively.

Multi‐level anticounterfeiting techniques integrate diverse anticounterfeiting technologies, employing multi‐layered structures, multiple modes, or multiple responses to bolster product security and thwart counterfeiting attempts.^[^ ^22^, ^23^, ^24^, ^25^, ^26^ ^]^ In such systems, anticounterfeiting information is intricately nested in various layers, requiring distinct decoding means to access the information. PUFs offer a distinctive encryption method that can be combined with other security methods, such as quick response (QR) codes and security inks, to achieve a higher level of security.^[^ ^27^, ^28^, ^29^ ^]^ Furthermore, leveraging the multiple and unique physical properties of random seeds (e.g., Raman, photoluminescence, scattering, fluorescence lifetime, etc.) facilitates the construction of multi‐layer PUFs to achieve high complexity and exponentially increased coding capacity.^[^ ^30^, ^31^ ^]^ Additionally, integrating different types of PUFs, such as optical‐based and morphology‐based PUFs, allows for joint verification of label authenticity, enhancing security while overcoming specific authentication limitations (e.g., reliance on large optical devices), thus broadening the scope of PUF applications.^[^ ^32^, ^33^, ^34^ ^]^ Nonetheless, crosstalk is prone to occur between signals from different types of PUFs, detrimental to the authentication accuracy of PUFs. Meanwhile, PUFs are expected to be complementary yet independent of each other to enhance the damage resistance of PUF labels, i.e., one PUF being damaged will not affect the integrity of the others, thus ensuring the overall reliability of the PUFs. Hence, achieving crosstalk‐free integration between different PUFs remains a challenge.

To address this problem, a strategy of integrating PUFs at different scales emerges as a promising option. Employing distinct authentication methods to verify PUF authenticity at various scales liberates them from the constraints of specific authentication devices. The phase separation structure, resulting from the thermodynamic instability of immiscible substances, offers a unique random pattern applicable to PUFs at the hundred‐micron scale.^[^ ^19^ ^]^ Notably, the blend of silk fibroin (SF) and polyvinyl alcohol (PVA) presents a common phase separation system, yielding a random morphology with additional biocompatibility.^[^ ^35^, ^36^ ^]^ This feature broadens the application scenarios of PUFs, encompassing biomaterials anticounterfeiting^[^ ^37^, ^38^ ^]^ and bioelectronic anticounterfeiting.^[^ ^39^, ^40^, ^41^ ^]^ Furthermore, diamonds can be used as ideal random seeds for PUFs due to their excellent physicochemical stability and distinctive response signatures, including Raman response, scattering, various photoluminescent color centers (e.g., nitrogen‐vacancy (NV) centers, silicon‐vacancy (SiV) centers, etc).^[^ ^22^, ^42^, ^43^, ^44^, ^45^ ^]^ Additionally, the abundant surface groups and good biocompatibility of nano‐ or micro‐sized diamonds facilitate their dispersion in polymers, promising robust information security as small‐scale PUFs. These materials and their unique properties fulfill the key requirements for creating PUF labels with different scales.

**Figure 1:**
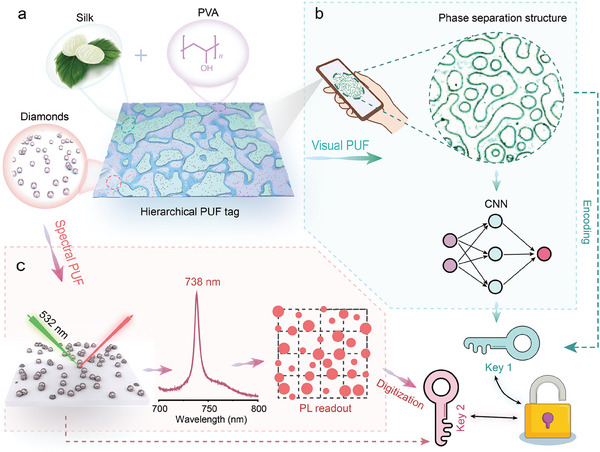
Schematic illustration of the hierarchical physical unclonable tags. a) The hierarchical PUF tag renders visual PUF and spectral PUF through SF/PVA phase separation and PL emission of SiV diamonds. b) The random phase separation structure generated by silk and PVA was utilized as the feature pattern of the visual PUF, and the convolutional neural network (CNN) was implemented to achieve rapid recognition and authentication. c) Random distribution of SiV diamond luminescence generates the spectral PUFs.

In this study, we present a novel hierarchical PUF system based on stochastic phase separation morphology derived from SF/PVA blend, in conjunction with the random distribution of SiV diamonds (**Figure** **1**a). The phase separation occurs spontaneously when the two polymer solutions, PVA and SF, are mixed, leading to inherent uncontrollable metastability that gives rise to random patterns utilized for information encryption. The information stored in these morphology features can be easily accessed using an optical microscope or a smartphone under oblique light, followed by authentication using customized algorithms (Figure 1b). To enhance authentication efficiency, a convolutional neural network (CNN) model is constructed specifically to classify the phase separation patterns based on classifiable features. Subsequently, a search and compare algorithm is executed within the qualified database. Additionally, by integrating microdiamonds into morphology code, a more advanced approach for data encryption is achieved, i.e., hierarchical encryption. This process exploits the photoluminescence (PL) intensity of the SiV centers of the diamond particles to enable high‐capacity optical coding, while Hamming distance and cross‐correlation functions are employed to authenticate the optical PUF (Figure 1c). The hierarchical PUF system exhibits remarkable efficiency in authentication, is free from crosstalk, and boasts outstanding reliability. By implementing PUF encoding at different levels, our system ensures secure authentication without interference between the levels. Furthermore, the multivariate and multi‐scale coding verification system enables a high coding capacity with a low bit error rate, significantly broadening the applications of hierarchical PUFs. Overall, our proposed hierarchical PUF system showcases substantial promise as a robust and efficient method for secure information encryption, offering new perspectives for a wide range of practical applications.

## 2 Results and Discussion

### 2.1 Design and Fabrication of Hierarchical PUFs

**Figure 2:**
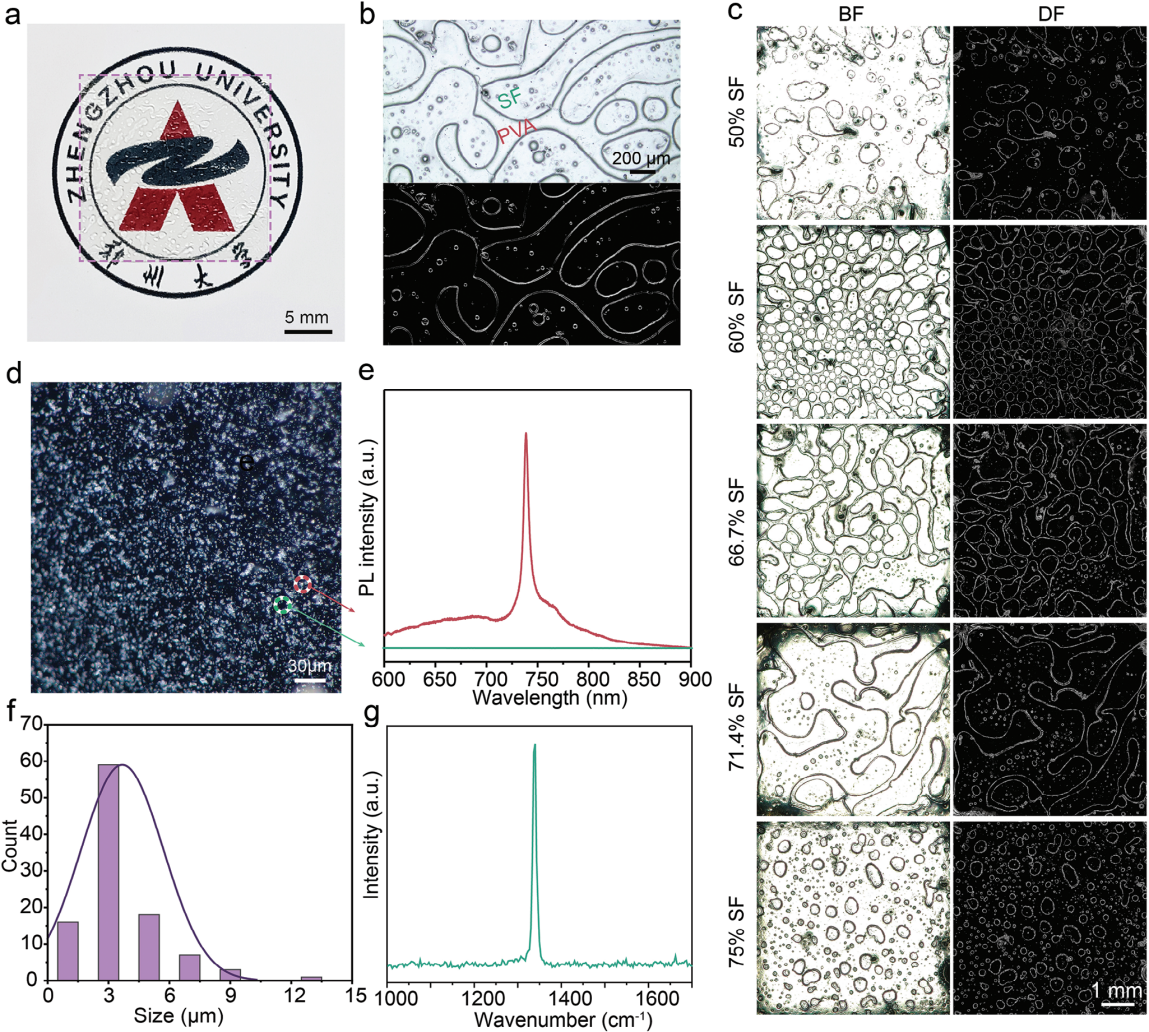
Characterization of the hierarchical PUF tags. a) Photograph of Hierarchical PUF labels. b) Typical bright‐field and dark‐field images of a hierarchical PUF tag, with intricate bicontinuous PVA/SF patterns clearly visible. c) Evolution of pattern morphology under different SF weight fractions. d) Dark‐field image of randomly distributed diamonds. e) PL spectra of two different positions with and without SiV diamonds, represented by the red and green circles in (d), respectively. f) Particle size distribution of the SiV diamonds. g) Raman spectra of the SiV diamonds.

The fabrication of the hierarchical PUFs system involves elaborate controlling of the phase separation morphology of the polymer blend and the dispersion of the SiV diamonds. The accurate reading and information entropy of the PUF keys are directly related to these controlled factors. **Figure** **2**a shows the optical image of a hierarchical PUF label with a visible phase separation contour and excellent transparency. The hierarchical PUF labels can be easily customized into various shapes to accommodate diverse applications, including design trademark customization or classification labels.

To create these labels, we introduced randomly oriented bicontinuous patterns in the diamond/SF/PVA blend films through spinodal decomposition of the unstable SF/PVA blend, as shown in Figure 2b. The bicontinuous mode offers a width greater than 100 µm, which can be captured with commercially available imaging sensors, including most smartphones. Interestingly, some spherical particles of random size are observed within the patterns, adding complexity to the anticounterfeiting mode. Raman characterization reveals a distinct spectral fingerprint of SF in regions with more spherical particles (Figure S1, Supporting Information). These spherical particles correspond to PVA domains trapped within SF domains, as previously reported in the phase separation of SF/PVA.^[^ ^36^ ^]^ The excellent light scattering performance at the phase separation interface renders the bicontinuous contour features more apparent in the dark field, facilitating efficient and rapid extraction of these features for encoding. Consequently, these bicontinuous contour features serve as the basis for digitizing the fast readouts of our hierarchical PUF labels.

Furthermore, by adjusting the ratio of SF and PVA, we can regulate the phase separation morphology and create different pattern morphologies in the SF/PVA blends. Figure 2c shows the typical SF/PVA blend patterns with different SF weight fractions. At a silk weight fraction of 66.7%, a uniform random bicontinuous pattern forms, while deviations from this value result in patchy or inhomogeneous discrete phases. Actually, the formation of phase separation patterns will be affected by multiple factors, including concentration, temperature, pressure, and so on (Figures S2 and S3, Supporting Information). The modulation of these pattern morphologies occurs within a narrow range of weight fractions, making it challenging to imitate PUF labels.

To enhance anticounterfeiting patterns, SiV diamonds were incorporated into the polymer blends. Figure 2d illustrates the diffuse and irregular distribution of tiny SiV diamonds in the PUF label. The unique near‐infrared (NIR) emission characteristics of SiV provide high contrast and a distinctive optical response, enabling SiV diamonds to function as random seeds for PUF tags. The representative optical responses of the SiV diamond‐based PUF are shown in Figure 2e, where the spots with diamond exhibit a sharp and intense spectral response at 738.5 nm (red circle in Figure 2d), whereas those without diamond exhibit a barely perceptible optical response (green circle in Figure 2d). In practice, the response intensity of each bit in PUF varies due to variations in diamond size and the fluctuation of the focused spot during measurement, which facilitates multilevel coding. These SiV diamonds exhibit a size distribution ranging from 1 to 10 µm, as shown in Figure 2f. The variation in size and shape enhances the chaotic nature of the PUF (Figure S4a, Supporting Information).

Figure 2g shows the Raman spectrum of SiV diamond with a sharp peak at 1332 cm^−1^ corresponding to the sp^3^ band configuration of the carbon atoms. The X‐ray diffraction (XRD) patterns of SiV diamonds reveal a narrow full width at half maxima (FWHM), demonstrating the excellent crystallinity of polycrystalline diamonds (Figure S2b, Supporting Information). Combining the stable optical properties of SiV under continuous laser irradiation shown in Figure S5 (Supporting Information), the PUF based on SiV diamonds enables stable repeat acquisitions of the same label. Additionally, the SiV diamonds are mainly dispersed in the bicontinuous phase, as evidenced in Figure S6 (Supporting Information). Unclonable bicontinuous patterns can be observed on the hundred‐micron scales and unclonable diamond distribution patterns appear on the tens‐of‐micron scales as the images are progressively enlarged. It is worth noting that the inclusion of diamonds causes no adverse interference with the capture and extraction of bicontinuous phase contours due to the significant difference in size. Meanwhile, the high transmittance of the bicontinuous phase ensures easy readout of PUF based on the SiV diamonds (Figure S7, Supporting Information), achieving an independent distribution of random patterns at different scales. We hereto refer to the PUF based on phase separation patterns as visual PUF, and the PUF based on PL mapping patterns as spectral PUF. This hierarchical PUF we propose incorporates separate unclonable patterns at different scales, exponentially increasing the cryptographic capacity and complexity of the PUF. These separate random patterns can be used individually for authentication or in combination, as needed. Moreover, having random patterns at various scales enhances the damage resistance of PUF tags, if one pattern fails, another pattern remains available for authentication, thereby ensuring the reliability of the PUF labels. These designs ensure that the hierarchical PUFs are more practical and reliable for various anti‐counterfeiting applications compared to other multi‐level PUFs (Table S1, Supporting Information).

### 2.2 The Recognition and Authentication of Visual PUFs

**Figure 3:**
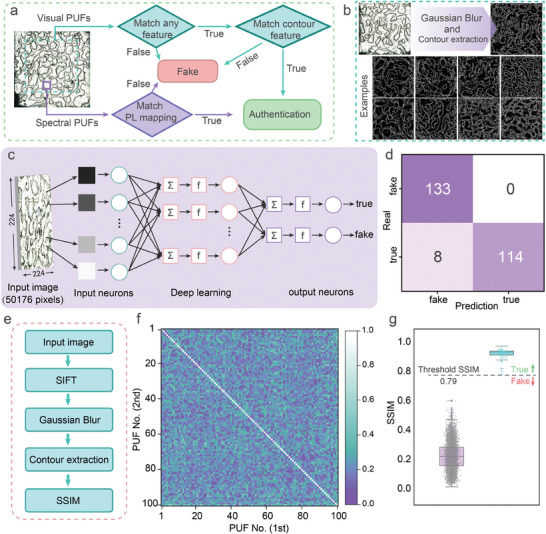
Readout and authentication of hierarchical PUF tags. a) Concept schematic of hierarchical PUF authentication. b) Description of the contours extracted from the initial images and some examples. c) The flow of the CNN‐based PUF authentication process. d) Confusion Matrix obtained from CNN. e) Flowchart of the authentication of visual PUFs. Heat maps f) and box plots g) of SSIM values extracted from visual PUF patterns.

Based on the distinctive pattern features of our hierarchical PUFs, we have devised a hierarchical authentication protocol, as depicted in **Figure** **3**a. The patterns at different scales of the PUF can be discerned based on their eigen morphologies. Consequently, different algorithms are exploited to authenticate the tags according to the distinct patterns of the PUF. For visual PUF, random patterns are captured using a commercial imaging sensing module. These collected images undergo filtering by a fast classification algorithm utilizing CNN. Subsequently, the filtered labels are compared with the dataset and authenticated by matching the structural similarity index (SSIM), a metric that ranges from 0 to 1. On the other hand, for the spectral PUF, mapping datasets are assigned by setting a threshold, where values above the threshold are assigned 1, while the values below it are assigned 0 to create the binary code. Authentication is then performed by comparing the Hamming distances of the binary codes. Furthermore, the datasets can be divided into equal intervals using multiple thresholds, resulting in a multi‐decimal code for subsequent authentication. To mitigate the interference of the acquisition environment, the initial images are uniformly subjected to an initial model correction procedure to achieve the optimum accuracy.

Figure 3b illustrates examples of the correction procedure applied to the visual PUF acquisition, confirming the effectiveness and generality of the procedure. The morphology of the resulting patterns demonstrates a clear difference, reaffirming the suitability of the diamonds/SF/PVA blends as PUFs. To further assess the complexity of the patterns generated based on phase separation, we calculated the 2D information entropy (2D entropy) and the fractal dimension. Figure S8a,b (Supporting Information) presents the values of the pattern entropy and fractal dimension obtained based on the visual PUF, both showing a narrow distribution, suggesting that the pattern formation relies on the same physical foundation. The extremely narrow distribution of the fractal dimension could be a criterion for evaluating the degree of visual PUF contouring. Only tags with values of the fractal dimension within the restricted range can be used properly. Moreover, the patterns maintain a uniform level of complexity while ensuring the randomness of the labels, exhibiting high intra‐category homogeneity, which allows for fast recognition using deep learning (DL) algorithms.

We further analyzed these patterns for categorization using a CNN, as shown in Figure 3c. As a proof of concept, a representative dataset of 433 patterns was extracted for the progressive training model, with 178 patterns (96 labeled “fake” and 82 labeled “true”) as the training set and a further 255 patterns (133 labeled “fake” and 122 labeled “true”) as the test set. All patterns were uniformly resized to 224 × 224 pixels prior to being fed into the CNN to learn features to improve operational efficiency. The model was applied to the test set for recognition after 200 iterations of progressive training, with a recognition accuracy of 96.9% (Figure 3d). Meanwhile, the test set containing 255 patterns can be authenticated with high confidence within 1 min, which greatly improves the efficiency of PUF authentication. However, despite the high recognition rates achieved by CNN, a certain gap still exists between the learning rate and the test rate, which can be attributed to substandard patterns or a degree of overfitting (as evidenced by examples shown in Figure S9, Supporting Information). Furthermore, while the CNN provides probabilistic guesses that are efficient for image classification, they may not be sufficiently convincing for accurate authentication, especially in terms of accurate information verification of individual labels.

To achieve accurate authentication of patterns, we have designed a recognition algorithm that combines the traditional scale‐invariant feature transform (SIFT) image alignment algorithm with pattern contour extraction. Figure 3e depicts the flowchart for SSIM validation. In practice, PUF reads are inevitably subject to shifts, rotations, and other variations, which can impede the accurate authentication of PUFs. To address this, our approach involves locating key points in the images, determining the feature vectors, and establishing spatial mapping relationships, to achieve the image alignment through a SIFT image alignment algorithm. Then, the images are recognized using SSIM after a uniform process of Gaussian blurring and contour extraction. Practically, this approach serves as a universal design strategy for various morphology‐based security labels. Figure S10 (Supporting Information) demonstrates the authentication process of a visual tag captured by a smartphone, where images with different sizes are readily authenticated after being processed by the recognition algorithm. Benefiting from the implementation of the SIFT image alignment algorithm, the reading of PUF tags escapes the constraints of the capture device and the majority of the external factors such as rotation, magnification, etc., as shown in Figure S11 (Supporting Information), thus facilitating the practical application of visual PUF tags.

Figure 3f showcases a cross‐comparison of a sample set consisting of 100 independent labels, yielding a total of 4950 (^100^C_2_) challenge‐response pairs (CRPs). The SSIM pairwise comparison diagram clearly indicates that identical labels exhibit high SSIM values relative to those between different labels, thereby demonstrating the excellent uniqueness of the visual PUFs. Based on the statistical classification of the box plot of SSIM, we allocate the threshold for SSIM authentication as 0.79 (Figure 3g). Labels above this threshold are recognized as true, otherwise as fake. Two additional batches of PUF labels were manufactured and authenticated to verify the consistency of the PUF tags, as shown in Figure S12 (Supporting Information). All tags were successfully authenticated with the authentication threshold being set to 0.79, demonstrating the excellent consistency and reliability of the PUF tags. Consequently, a two‐step authentication mechanism has been successfully developed, which significantly reduces the overall authentication time of the labels while maintaining a high level of authenticity. This strategy of fast classification combined with accurate authentication is expected to strike a balance between large databases and short authentication times, making it widely applicable. Moreover, the low‐barrier capture approach and the straightforward modular verification procedure facilitate the rapid adoption of PUFs in practical applications.

### 2.3 The Key Generation and Authentication of Spectral PUFs

**Figure 4:**
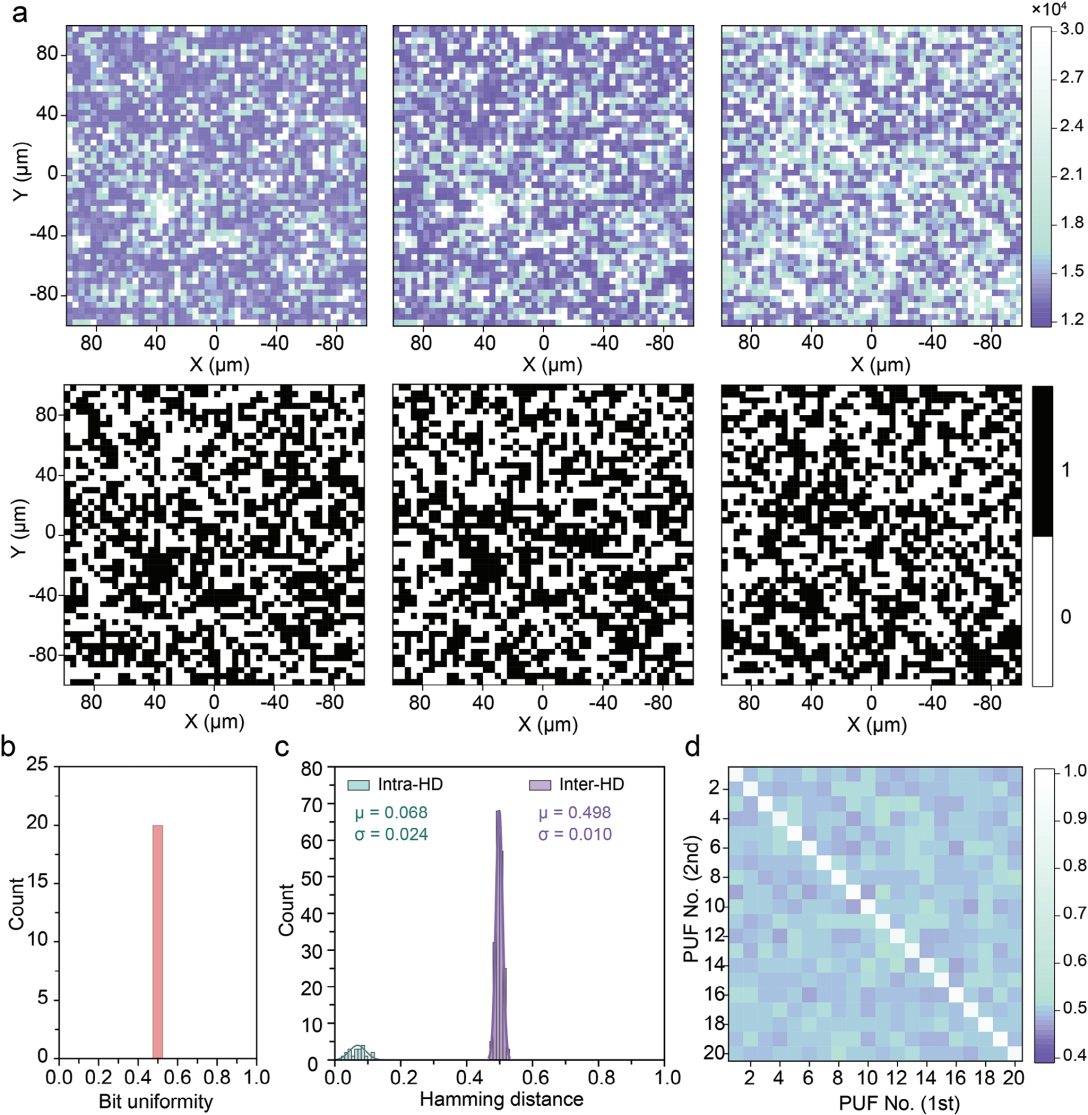
Readout and authentication of spectral PUF with binary coding. a) PL mapping images and the corresponding binary encoding matrix of the spectral PUF with a resolution of 50 × 50 pixels for the first (left), second (middle) measurement, and another PUF with the same measurement (right). b) Bit uniformity calculated on the basis of 20 different spectral PUFs. c) Statistical distribution of inter‐device Hamming distances (HD) and intra‐HDs of 20 different spectral PUFs, with the mean value of inter‐HDs and intra‐HDs of 0.498 and 0.068, respectively. d) Pairwise comparisons of 20 different PUF tags with binary encoding at each pixel for the first and second measurements.

To create spectral PUFs, an area of 200 × 200 µm^2^ within the bicontinuous phase was selected to serve as a spectral label. The bicontinuous phase features are utilized as markers to facilitate consistent testing of the same area to ensure reliable authentication of the PUF labels (Figure S13, Supporting Information). **Figure** **4**a presents the binary coding matrix derived from repeated measurements of the same PUF label, as well as different labels, with a resolution of 50 × 50 pixels. It is evident that repeated measurements of the same label exhibit a high degree of similarity, while different labels show significant differences from each other.

To ensure the practicality of the spectral PUFs, the benchmark performance of the PUF was further systematically evaluated. Bit uniformity, which estimates the probability of the digits 0 and 1 appearing in the PUF code, ideally should be 0.5. In our case, the bit uniformity for 20 spectral PUFs is estimated to be 0.50002, indicating an exceptional level of bit uniformity (Figure 4b). Hamming distances (HD) are introduced to measure the number of mismatching bits between two binary codes as a basis for investigating the uniqueness and repeatability of PUF codes. Uniqueness is assessed by calculating the inter HD, defined as the HD between PUF codes obtained from two different tags under the same challenge, to determine whether each PUF code can be unambiguously identified. The ideal inter HD between different PUF codes should be 0.5, signifying a complete lack of correlation between labels. A total of 190 pairs (20 × 19/2) of CRPs were calculated for the normalized inter‐HDs and plotted as histograms (Figure 4c). The histogram shows a well‐fitted Gaussian distribution with a central value of 0.4987 and a standard deviation of 0.0101, confirming the uniqueness of the spectral PUFs.

The repeatability of the labels was evaluated using intra HD, which is defined as the HD between PUF codes obtained under the same challenge for the same label. For the 20 different labels, the estimated intra‐HD realized a low mean value of 0.0688 with a standard deviation of 0.0248, demonstrating good repeatability. Also, the false negative rate was further estimated based on inter and intra‐HD variables, yielding an impressively low value of 8.238 × 10^−15^ (Figure S14, Supporting Information), indicating that the initial authentication of the spectral PUF had an extremely low probability of failure. Figure 4d depicts the similarity index plot for the pairwise comparison of 20 different spectral PUF tags. The distinguishable diagonal and nondiagonal elements reaffirm the uniqueness of each spectral PUF, enabling reliable and repeatable authentication. Besides, the similarity indices on the diagonal are all above 89%, and this value can be used as a criterion for PUF tag authentication (e.g., above this value, authentication succeeds; otherwise, it fails). Randomness is a fundamental property of PUFs that ensures their unpredictability. To verify the randomness of the digitized keys extracted from the spectral PUFs, the randomness test was conducted using the suite SP 800–22 developed by the National Institute of Standards and Technology (NIST). All seven NIST random tests were successfully passed by using 96 128‐bit PUF codes extracted from the spectral PUFs, as shown in **Table** **1**. This confirms the randomness of the spectral PUFs, indicating that spectral PUFs are unpredictable and difficult to replicate.

**Table 1:** Summary of the randomness tests of binary sequences generated from hierarchical PUFs.

^a)^NIST statistical test	^b)^ *p*‐value	Proportion	Result
Monobit	1.0	96/96	PASS
Frequency within block	0.390907317	94/96	PASS
Runs	0.367200983	91/96	PASS
Longest‐run ones in a block	0.479003574	94/96	PASS
Serial	0.492054873	95/96	PASS
	0.50347138	95/96	PASS
Approximate entropy	0.490527523	94/96	PASS
Cumulative sums	0.275709635	94/96	PASS
	0.625507036	96/96	PASS

^a)^ Results of NIST test for PUF construction. The text dataset is performed using 96 sequences of 128 bits collected from 30 different PUFs. The bitstream is only considered random if the *p*‐value is ≥ 0.0001.

^b)^ If the pass rate exceeds the minimum rate (>91/96) for each test, it is considered a pass.

**Figure 5:**
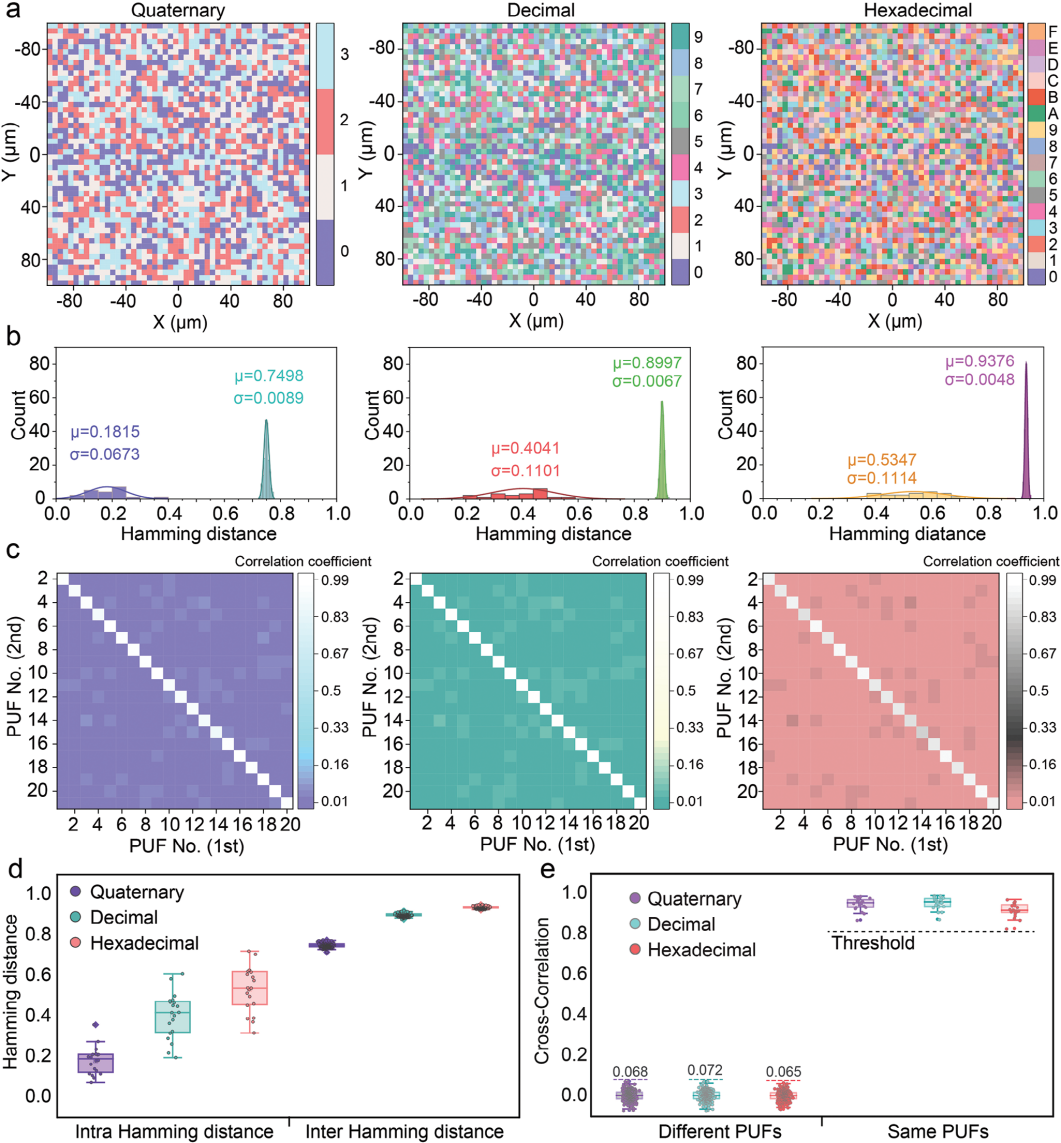
Encryption and authentication of spectral PUFs with M‐ary encoding. a) M‐ary matrices in quaternary (left), decimal (middle), and hexadecimal (right) encoding of the spectral PUF with a resolution of 50 × 50 pixels. b) Statistical distribution of inter‐device HDs and intra‐HDs of 20 different spectral PUFs in quaternary (left), decimal (middle), and hexadecimal (right) coding. c) Pairwise comparisons as a cross‐correlation function of 20 different spectral PUFs for the first and second measurements. d) Box plot of Hamming distances of spectral PUFs with M‐ary encoding. e) Box plot of cross‐correlation values of spectral PUFs with M‐ary encoding.

To enhance the complexity of PUF encryption, M‐ary coding (e.g., quaternary, decimal, hexadecimal) can be introduced, leveraging the random and diverse SiV PL intensity of each pixel in the fluorescent image. **Figure** **5**a illustrates the M‐ary encoding matrix for PL mapping of the same label, demonstrating a chaotic and intricate distribution. In order to investigate the feasibility of M‐ary encoding, an evaluation was conducted using HD, and the corresponding results are shown in Figure 5b. The histograms of the inter HD, based on quaternary, decimal, and hexadecimal coding, are well‐fitted by Gaussian distributions with mean values of 0.7498, 0.8997, and 0.9376, respectively, which are remarkably close to their ideal values of 0.75, 0.9, and 0.9375. This signifies that the labels maintain excellent uniqueness even under M‐ary encoding.

It is noteworthy that the information entropy of PUF codes varies with changes in the code element length, which provides a way to achieve a high coding capacity of PUFs. Figure S15 (Supporting Information) illustrates the information entropy of the PUF codes obtained by encoding based on different code element lengths, which increases as the code element length increases. Meanwhile, the information entropies obtained are all close to the ideal value, signifying the enormous chaos of PUF codes and their inherent unpredictability. The coding capacity can be estimated to exceed 1.584 × 10^12041^ (16^10000^) according to the coding pattern (Figure S16, Supporting Information). Following the experimental approach of this experiment, the encoding capacity can be exponentially increased by expanding the capture size of the spectral PUF tags. However, it's important to note that the current encoding capacity is already sufficient for most authentication applications.

To evaluate the repeatability of the PUF labels with M‐ary encoding, the intra‐HD was calculated. However, the obtained values deviated significantly from the ideal value of 0 and were exacerbated with increasing code element length. This discrepancy could lead to a larger bit error rate (BER) during PUF verification. During data capturing, minor shifts in the data are always inevitable due to factors such as light source decay, moving stage deviations, or focus point variations. Nevertheless, as the code element length increases, the resistance to small errors in code element assignment degrades, resulting in an increased occurrence of nonzero values in the HD calculations. Since the HD is commonly defined as the number of different characters in the corresponding positions of two strings with the same length, it is no longer suitable for verifying the repeatability of M‐ary encoded PUFs. To address this issue and assess the repeatability of the M‐ary encoded PUFs more effectively, the cross‐correlation function was introduced, which is commonly employed to investigate the degree of linear correlation between two codes, with values ranging from −1 to 1. A correlation coefficient close to 1 or −1 indicates a strong correlation between the two codes, while a value close to 0 indicates no deterministic correlation and suggests randomness. Figure 5c presents the results of the PUF authentication using the cross‐correlation function, with significant variation between diagonal and nondiagonal regions, indicating that the M‐ary encoded PUFs achieve a highly repeatable authentication. Furthermore, the diagonal areas all exhibit values around 0, confirming the uniqueness of the PUF labels.

To make a more explicit comparison between the Hamming distance and the cross‐correlation function applied to the M‐ary coded PUFs, the distributions of their values were statistically calculated using box plots, as shown in Figure 5d,e, respectively. For both authentication protocols, 20 different PUF tags were executed for each test, showing a significant improvement in the repeatability of the authentication protocol using the cross‐correlation function compared to the Hamming distance. This outcome confirms that the introduced cross‐correlation function is well applicable to the authentication process for M‐ary codes, providing a more reliable and accurate measure of repeatability. In real‐world usage, minor inaccuracies in raw data are inevitable due to equipment interference or environmental factors. The significant divergence in correlation coefficients allows for the adjustment of the verification threshold based on specific circumstances, ensuring a high rate of validation success. Furthermore, the comprehensive PUF multi‐ary encryption framework offers a more flexible and reliable anti‐counterfeiting solution.

### 2.4 Reliability of Hierarchical PUFs

The stability of hierarchical PUFs in various environments is further investigated to meticulously assess the true reliability of PUF tags. Figure S17 (Supporting Information) illustrates the performance of PUF tags at different temperatures. As the temperature increases, the visual PUF patterns show higher contrast, which facilitates the extraction of contours. The visual PUFs can be verified up to 150 °C, demonstrating acceptable thermal stability. However, when exposed to higher temperatures, the tags gradually degraded, with significant carbonization occurring at 200 °C, leading to label failure. The exceptional thermal stability of SiV diamond supports the favorable temperature resistance of spectral PUFs, which exhibit excellent repeatability over a range of 150 °C. Unfortunately, the detection of SiV emission at 200 °C was impeded by silk/PVA carbonization, leading to label failure. Similarly, the reliability of hierarchical PUFs under various humidity environments was evaluated, as shown in Figure S18 (Supporting Information). The hierarchical PUFs remained properly authenticated when exposed to less than 78% relative humidity (RH), yet failed in higher humidity environments or in water, due to the inherent hydrophilicity of silk/PVA. However, the water degradability of hierarchical PUF in conjunction with its excellent biocompatibility offers potential applications in drug security and implantable electronic anti‐counterfeiting.

## 3 Conclusion

In summary, multi‐level PUF labels that exploit random patterns generated by spontaneous phase separation and stochastic mappings generated by the disordered distribution of SiV diamonds have been developed. This achievement allows for effective anticounterfeiting at different scales. The fast recognition of visual PUFs based on the random pattern contours is accomplished using a complementary algorithm of CNN superimposed with SSIM, making it easy to implement and widely applicable. The capture and verification of random patterns can be accomplished on commercial portable devices such as smartphones, streamlining widespread practical adoption. Simultaneously, competitive crosstalk‐free spectral PUFs are effectively achieved by utilizing the stable luminescence of SiV diamond at a much smaller scale. Furthermore, the coding capacity of the spectral PUF can be further increased by using the M‐ary encoding method. Unlike increasing the coding capacity by elevating the mapping resolution, which would lead to a trade‐off between resolution and scan time, increasing the code element length is a more straightforward implementation. Customized authentication protocols enable high‐accuracy verification of M‐ary encoded PUFs, ensuring system reliability. This multi‐level, multi‐scale PUFs platform provides a reliable and high‐performance anti‐counterfeiting solution with promising applications in information protection and data storage.

## 4 Experimental Section

### Materials

Bombyx mori silkworm cocoons were purchased from Anguo Xufang Chinese Herbal Medicine Management Co. Ltd. Polyvinyl alcohol (PVA), calcium chloride (CaCl_2_), sodium bromide (NaBr), ammonium chloride (NH_4_Cl), sodium carbonate (Na_2_CO_3_), disodium hydrogen phosphate (Na_2_HPO_4_) and lithium bromide (LiBr) were purchased from Shanghai Maclean Company.

### Preparation of SF/PVA Blends

SF aqueous solution and PVA aqueous solution were separately prepared as in the previous work.^[^ ^46^ ^]^ Bombyx mori cocoons were first cut into small pieces and boiled in a 0.01 m Na_2_CO_3_ solution for degumming. The resulting sediments were then washed three times with deionized water. Next, the degummed silk fibroin (SF) was dissolved in a 9.3 mol L^−1^ LiBr solution at 60 °C for 4 h, and dialyzed with distilled water for 48 h to achieve the SF solution. For further processing, the SF solution was centrifuged at 10 000 rpm for 10 min and then concentrated to a final concentration of 20 wt.% using a dialysis bag covered with sucrose outside. PVA solution (10 wt.%) was obtained by dissolving 10 g PVA in 90 g deionized water and magnetically stirring at 96 °C for 2 h. Finally, SF aqueous solution (20 wt.%) and PVA aqueous solution (10 wt.%) were mixed in a ratio of 1:1‐3 to obtain the SF/PVA blends.

### Synthesis of Silicon Vacancy (SiV) Diamonds

SiV diamonds were obtained by the microwave plasma chemical vapor deposition (MPCVD) method on a silicon substrate. H_2_ and CH_4_ were used as carrier gases and sources respectively. The CH_4_/(H_2_ + CH_4_) gas ratio was 1–20%. The deposition time was 6–12 hours at a substrate temperature of ≈930 °C. The resulting samples were then treated using a 40% NaOH aqueous solution to remove silicon. After washing with deionized water, the samples were further ground in an agate mortar for 3 h. Then, the resulting samples were soaked in hydrofluoric acid for 12 h and later washed three times with deionized water to obtain irregular microdiamonds.

### Fabrication of the Hierarchical PUFs

SiV diamonds were dispersed into deionized water to give 5 wt.% suspension liquid and then dropped into SF/PVA blends to obtain diamond/SF/PVA hybrids. The diamond/SF/PVA hybrids were dropped onto the target object to dry naturally to form PUF tags. The PUF tags with different thicknesses were prepared by tuning the dosage of the hybrids (10 , 20, 30, 40, and 50 µL cm^−2^). A dose of 30 µL cm^−2^ was selected for routine use. The synthesis of PUF tags at different temperatures was finished on a heated bench.

### Characterization

The crystal structure of SiV diamonds was analyzed by X‐ray diffraction (XRD) patterns (X'Pert Pro, PANalytical, Netherlands). The size distribution of SiV diamonds was characterized by field emission scanning electron microscope (Auriga, Zeiss, Germany). X‐ray diffraction (X'Pert Pro, PANalytical, The Netherlands) patterns were used to analyze the crystal structure of SiV diamonds. The Raman measurement and the photoluminescence (PL) spectroscopy were carried out using a spectrometer of the SOL instrument (Confotec MR520, SOL instrument Ltd., Republic of Belarus) under the excitation of a 532 nm laser (50 mW, 20× objective lens). Exposure time is 0.1 s per pixel.

### Authentication of Hierarchical PUFs

For the visual PUFs, the optical images were collected using an optical microscope (DSX1000, Olympus Corporation, Japan) under bright field and dark field. Meanwhile, the visual PUFs were captured using a smartphone (Reno6 Pro, OPPO, China) for a practical authentication demonstration. Then, the images were processed with a custom Python program to extract and draw the contours of the bicontinuous phase formed by SF/PVA blends. To equalize the illumination, the original image was processed with a Gaussian blur to reduce peak/edge noise and lower the detail level. Next, a convolutional neural network (CNN) model was built to filter the visual PUF patterns. Similar to the previous work,^[^ ^47^ ^]^ the deep learning networks used in this paper are all based on PyTorch and run on the Jupyter Notebook. The CNN models are obtained by migration learning on top of a deep residual network 18 model. The computers used for deep learning were equipped with a CPU (Intel(R) Core (TM) i7‐10700), GPU (NVIDIA GTX 3060), RAM (16.0 GB), and HDD capacity (4 TB).

The passed PUFs were further authenticated by measuring the SSIM with the data in the database. All algorithms were developed using Python 3.9. The SIFT alignment process consists of several steps. First, the key points and descriptors of the image were detected using the SIFT detector from the OpenCV python package, and then the key points were matched and perspective transformed. The resulting images were cropped, Gaussian blurred, and greyed to obtain a grey histogram to select the threshold. After that, the image was binarized according to the threshold value, and the contour was extracted to obtain a binarized contour image. Finally, the images were compared using the compare_ssim function from skimage.metrics.

The spectral PUFs utilize the photoluminescence (PL) of SiV centers in diamonds for the readout of PUF tags, which was achieved using a confocal microscope system (Confotec MR520, SOL instrument Ltd., Republic of Belarus). The system utilized a 50× objective lens and a 532 nm laser with a power output of 50 mW as the excitation source. Each pixel was exposed for 0.1 s during imaging. Stray light was filtered out using a 532 nm long‐pass filter. The PL mapping with different resolutions (50 × 50 and 100 × 100 pixels) was obtained by varying the distance of the laser spot in a square area of 200 × 200 µm^2^. Repeat the measurement three times and calibrate the position before each measurement. Afterward, a MATLAB program was developed to digitize and authenticate spectral PUFs. The program extracts digital keys and analyzes digitized matrices. Initially, the raw PL response images were normalized by quantifying the intensity of the PL signals in each pixel. Then, a threshold was set to classify the detected responses. If the response value was below the threshold, it was assigned a value of 0. Otherwise, it was assigned a value of 1, which represents the code value. The Hamming distance between two data matrices was then calculated and compared to further authenticate the PUFs. The bit uniformity can be calculated using the following equation:

(1) $$Bit\;unifomity\;=\frac{1}{t}\;\sum_{n\;=\;1}^{t}K_{n}$$

where *K*_n_ is the *n*‐th binary bit in the PUF digitized keys and *t* is the key size.

The device uniqueness between any two PUF patterns can be defined as:

(2) $$Device\;uniqueness\;=\frac{2}{ss-1}\;\sum_{n\;=\;1}^{s-1}\sum_{m\;=\;n+1}^{s}\frac{\mathrm{HD}(K_{n},\;K_{m})}{t}$$

where *K*_n_ and *K*_m_ are *t*‐bit keys of the *n*‐th PUF device and the *m*‐th PUF device, respectively, and *s* is the total number of the PUF labels.

The readout reproducibility can be calculated as the following equation:

(3) $$Readout\;reproducibility\;=\sum_{i=1}^{t}\frac{\mathrm{HD}(K_{n},\;{K_{n}}^{\prime })}{t}\;$$

where *K*_n_ is the original *t*‐bit reference binary coding matrix and *K*_n_’ is a *t*‐bit binary coding matrix extracted from repeated measurements of the same PUF label.

For the M‐ary encoding, multiple thresholds (three, nine, and fifteen) were set to classify and assign values according to the actual condition. Nonfixed thresholds were adopted for encoding to ensure flexibility in encoding. The raw data was first sorted according to the size of the values, and then the raw data was uniformly distributed into N small subsets, where N is the number of code elements. Then the maximum value of each subset was extracted to be utilized as a threshold value. Finally, the raw data was encoded according to the obtained threshold. To rectify the bias of the Hamming distance in the M‐ary encoding system, a cross‐correlation function was introduced for the authentication of the M‐ary encoding system.

The cross‐correlation coefficient can be defined as:

(4) $$R_{xy}=\frac{cov\left(x,y\right)}{\left(std\left(x\right)*std\left(y\right)\right)}\;$$

Where *cov(x,y)* denotes the covariance of x and y; *std(x,y)* denotes the standard deviation of x and y.

The similarity index was calculated as a function:

(5) $$\mathrm{Simi}\mathrm{lari}\mathrm{ty}\;\mathrm{index}\;=\;1-\frac{\sum_{i=1}^{t}\sqrt{{\left(K_{i}-K_{i}^{'}\right)}^{2}}}{t}\times 100\%$$

where *K* is the original *t*‐bit reference binary coding matrix and *K’* is a *t*‐bit binary coding matrix extracted from repeated measurements of the same PUF.

The entropy of the bit distribution is calculated as a function:

(6) $$H\;=\;-\sum_{i\;=\;1}^{n}\left(p\left(i\right)*\log_{2}\left(p\left(i\right)\right)\right)$$

where *H* is the information entropy and *p(i)* is the probability of each category in the data. *n* is the number of values taken by the random variables. For binary, quadratic, decimal, and hexadecimal codes, the values of n are 2, 4, 10, and 16 respectively. The range of values of *H* is 0 ∼ log _2_(*n*). Therefore, the maximum *H*‐values for the M‐ary encoding are 1, 2, 3.322, and 4, respectively.

The fractal dimension is defined as:

(7) $$D\;=\lim_{\varepsilon \to 0}\frac{\log N\left(\varepsilon \right)}{\log\frac{1}{\varepsilon }}\;$$

where *N* indicates the number of measurement units, ε is the scaling factor.

### Reliability Test

For the visual PUF, the labels were annealed by placing them on a hot stage at different temperatures for 2 h followed by imaging. For the spectral PUFs, thermal reliability is performed in situ in the range of room temperature to 200 °C with a heating rate of 5 °C min^−1^ using a heating/cooling stage (T96‐S, LINKAM, UK). For humidity stability (RH), the humidity environment was provided by different saturated salt solutions of CaCl_2_, NaBr, NH_4_Cl, Na_2_CO_3_, and Na_2_HPO_4_ in closed glass vessels at room temperature (25 °C) to form RHs of 31%, 58%, 79%, 92%, and 98%, respectively. The tags were placed in different humidity environments and stabilized for 3 h before testing.

## Conflict of Interest

The authors declare no conflict of interest.

## Supporting information

Supporting Information

## Data Availability

The data that support the findings of this study are available from the corresponding author upon reasonable request.
