# Supplementary material for: Silicon Vacancies Diamond/Silk/PVA Hierarchical Physical Unclonable Functions for Multi‐Level Encryption
Source: Adv Sci (Weinh). 2024 Apr 4;11(23):2308337. doi: 10.1002/advs.202308337 (PMC11186112; doi:10.1002/advs.202308337)
Supplement: Supplementary file 1 — Supporting Information [file ADVS-11-2308337-s001.pdf]

ADVANCED SCIENCE
Open Access

Supporting Information

for *Adv. Sci.*, DOI 10.1002/advs.202308337

Silicon Vacancies Diamond/Silk/PVA Hierarchical Physical Unclonable Functions for Multi-Level Encryption

Fuhang Jiao, Chaonan Lin, Lin Dong, Xin Mao, Yi Wu, Fuying Dong, Zhenfeng Zhang, Junlu Sun, Shunfang Li, Xun Yang, Kaikai Liu, Lijun Wang and Chong-Xin Shan**

Supporting Information

Silicon Vacancies Diamond/Silk/PVA Hierarchical Physical Unclonable Functions for Multi-Level Encryptionv

Fuhang Jiao[1†], Chaonan Lin[1†], Lin Dong[1], Xin Mao[1], Yi Wu[2], Fuying Dong[1], Zhenfeng Zhang[1], Junlu Sun[1], Shunfang Li[1], Xun Yang[1], Kaikai Liu[1], Lijun Wang[1], and Chong-Xin Shan[1*]*

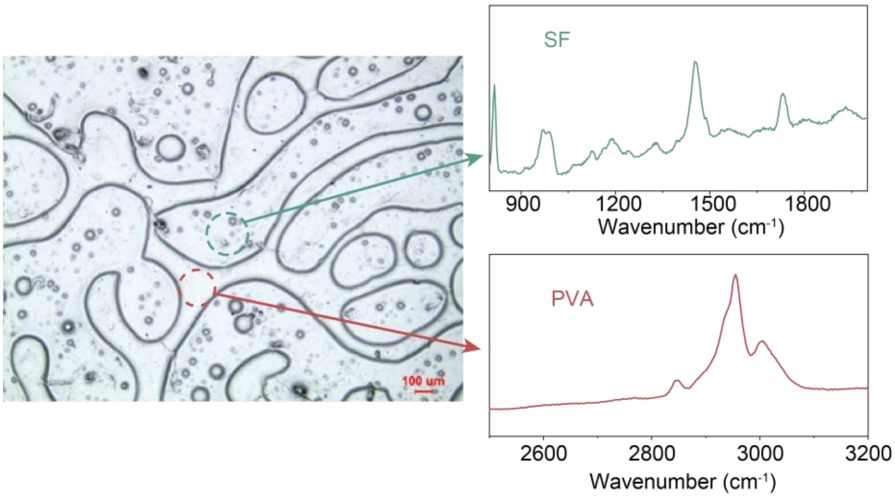

Figure S1. Raman spectra of different regions of visual PUF label based on phase separation

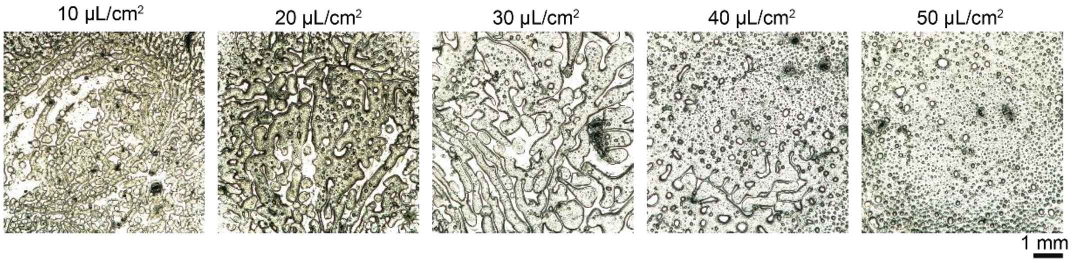

Figure S2. Evolution of pattern morphology with various dosage of the mixed solution.

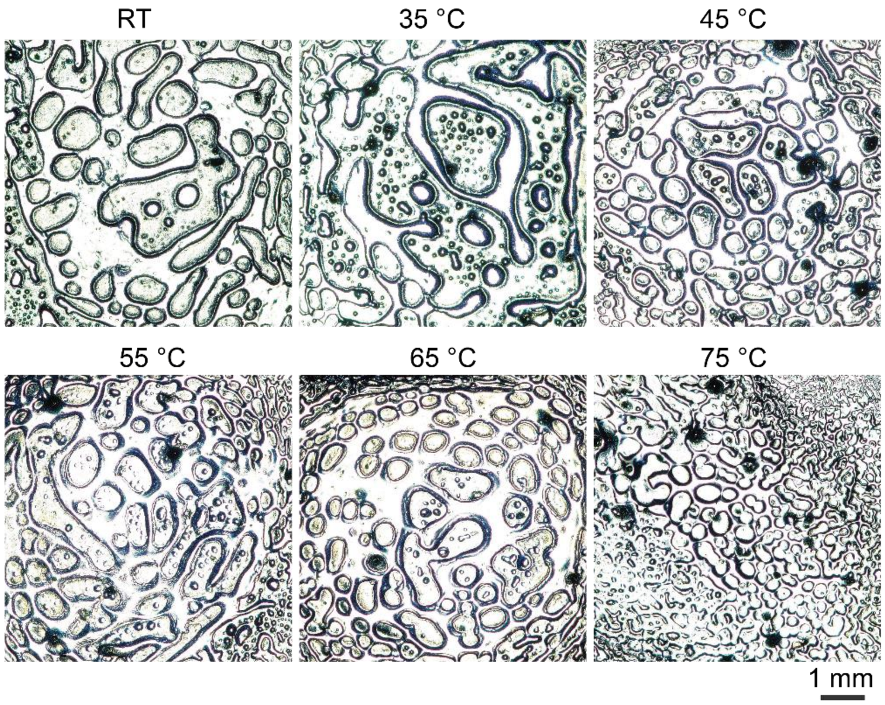

Figure S3. Evolution of pattern morphology drying at different temperatures.

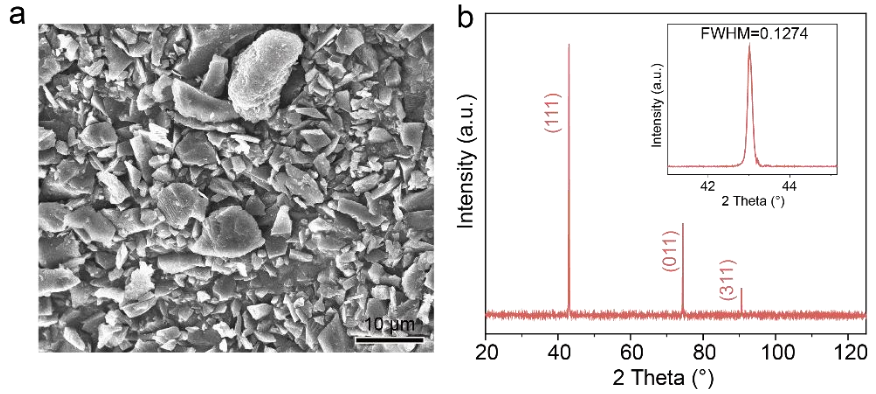

Figure S4. (a) SEM images and (b) XRD patterns of SiV diamonds.

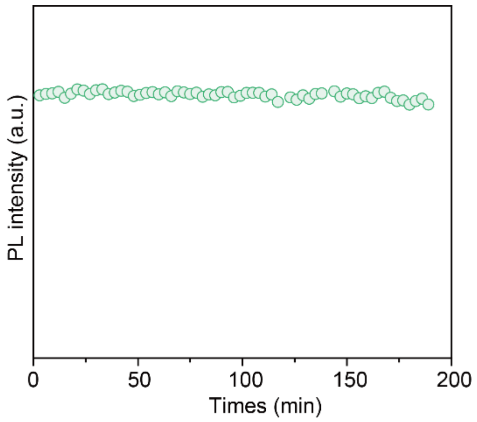

Figure S5. Photoluminescence stability of the SiV diamonds excited with a 532 nm laser.

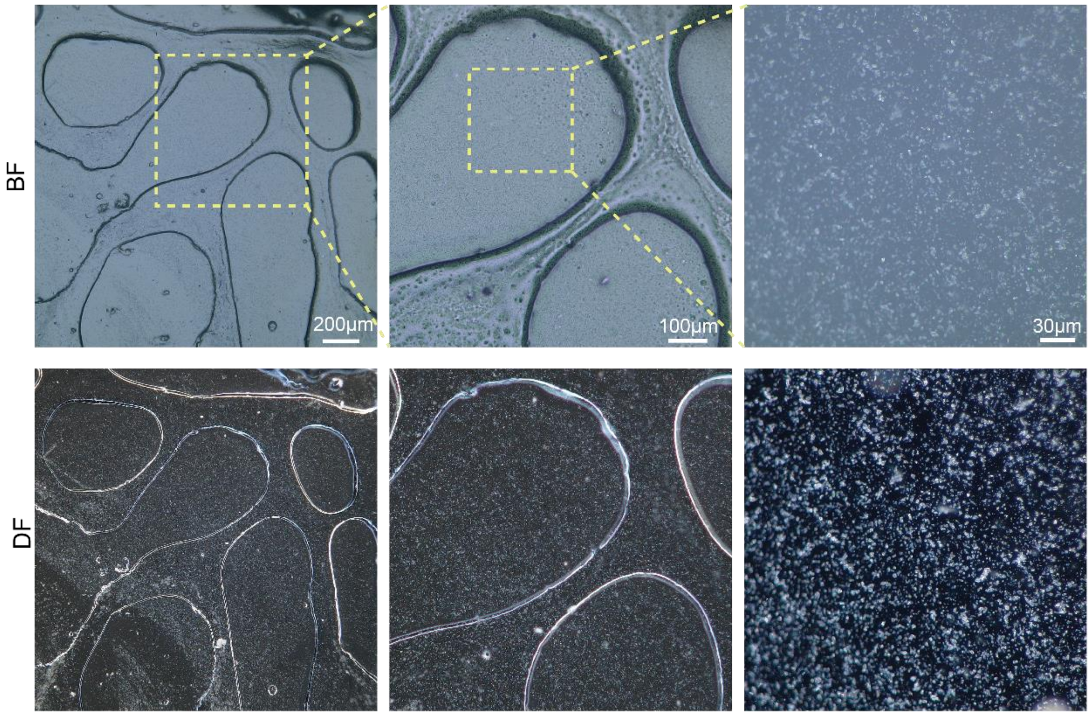

Figure S6. Stepwise magnification of optical microscopy images of the hierarchical PUF labels.

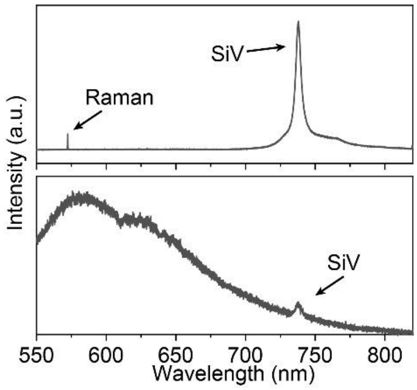

Figure S7. PL spectra of SiV diamonds with and without silk/PVA.

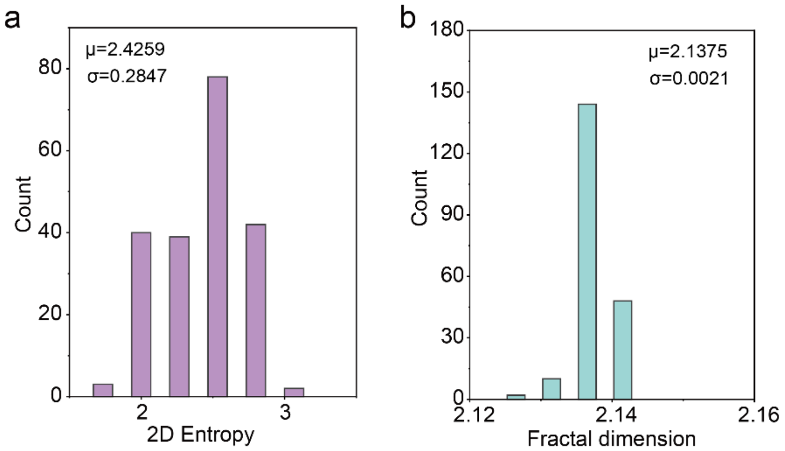

Figure S8. The 2D entropy and fractal dimension of the visual PUFs.

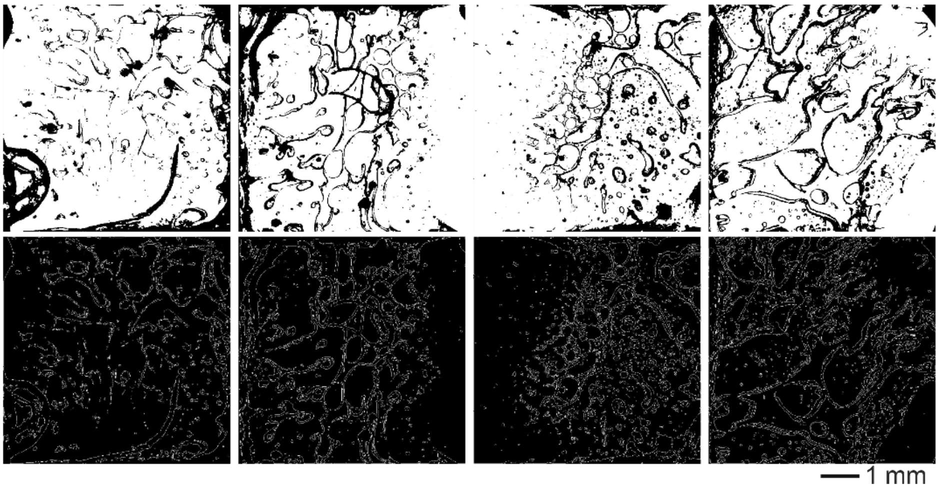

Figure S9. Some examples of the visual PUFs with substandard patterns or a degree of overfitting.

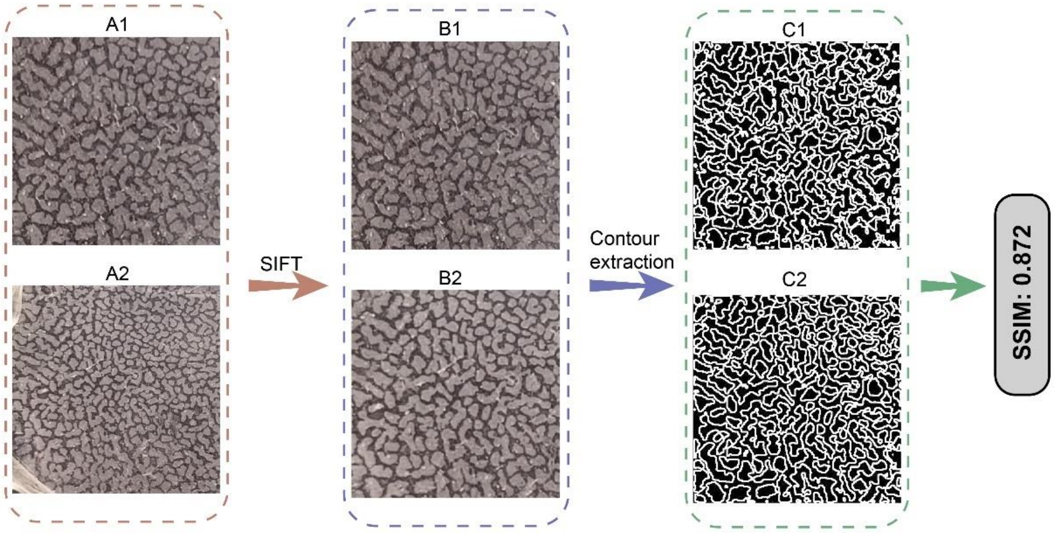

Figure S10. Readout and authentication of visual PUF captured by smart phone.

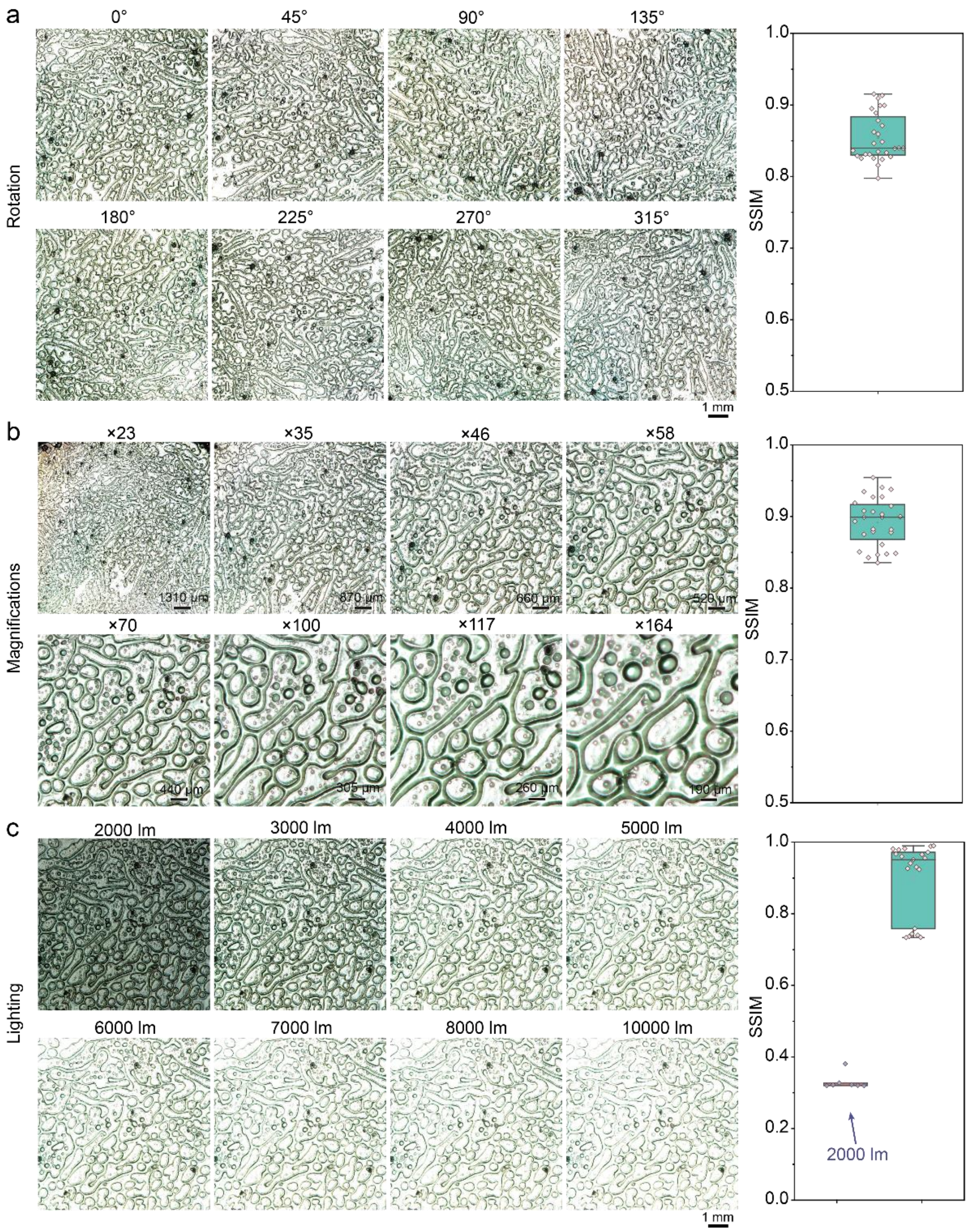

Figure S11. Authentication of visual PUFs captured under different conditions. (a)rotation, (b)magnifications and (c)lighting.

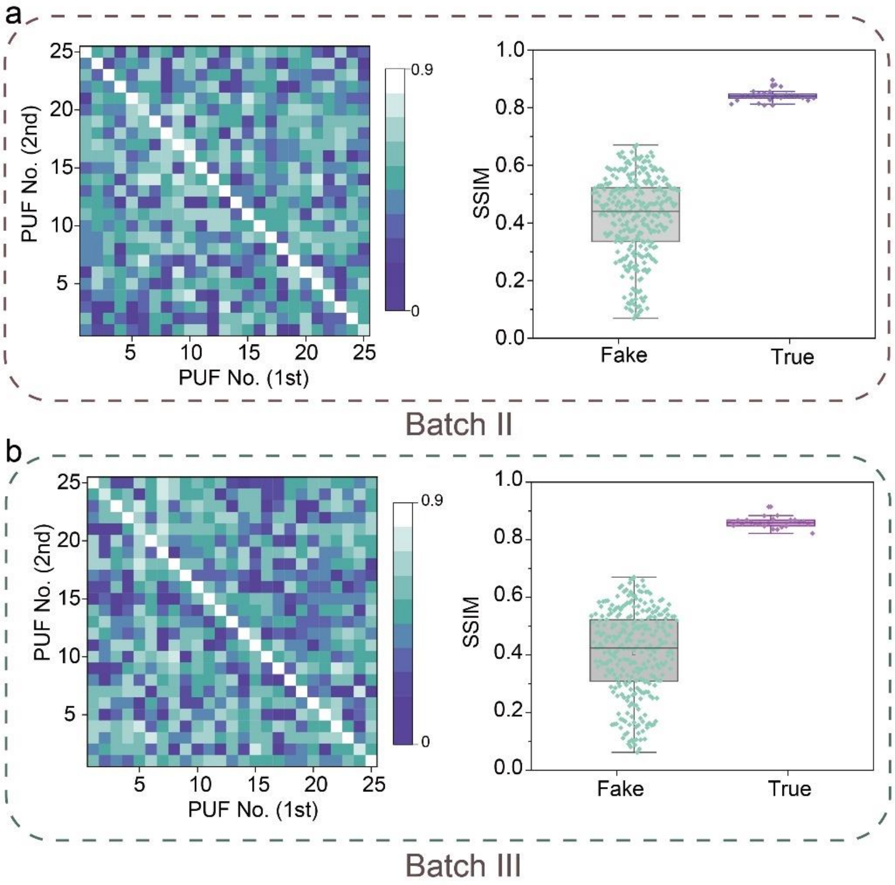

Figure S12. (a) Heat maps and box plot of SSIM values extracted from visual PUF patterns of the batch II manufacturing. (b) Heat maps and box plot of SSIM values extracted from visual PUF patterns of the batch III manufacturing.

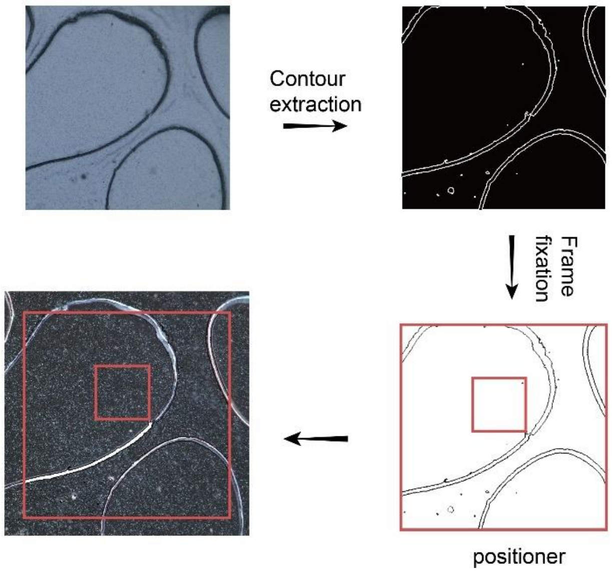

Figure S13. Schematic diagram of bicontinuous features as a localization framework for spectral PUF.

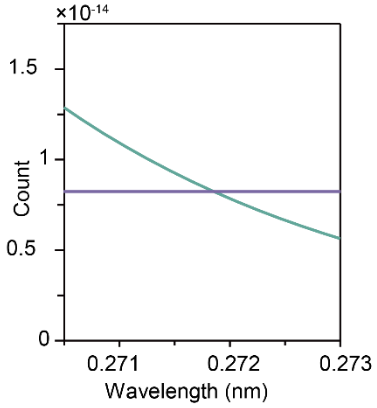

Figure S14. The false negative rates obtained from the intra- and inter-device variability with a cut-off of 0.2718 are 8.238×10^{-15}.

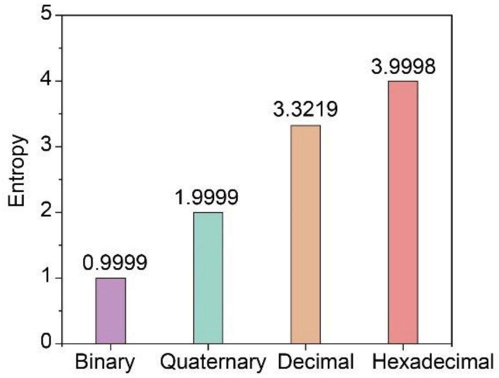

Figure S15. The entropy of PUF labels with M-ary encoding.

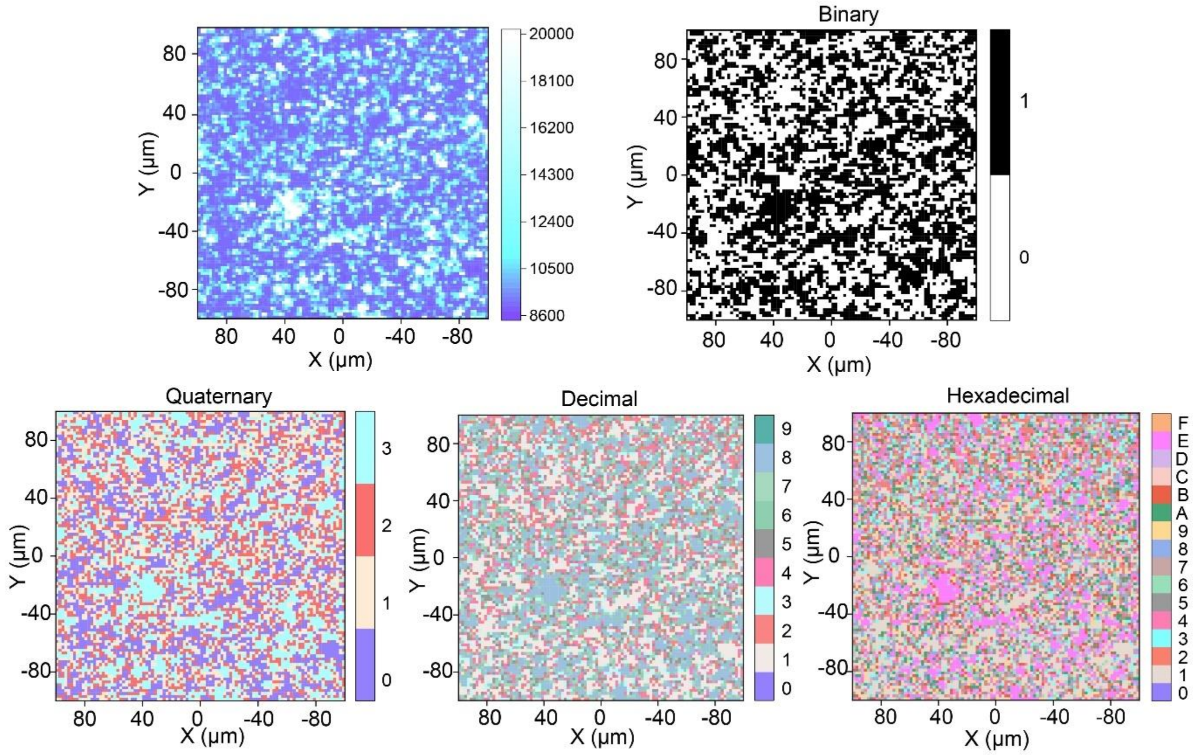

Figure S16. The M-ary encoded PUF label with 100 × 100 pixels.

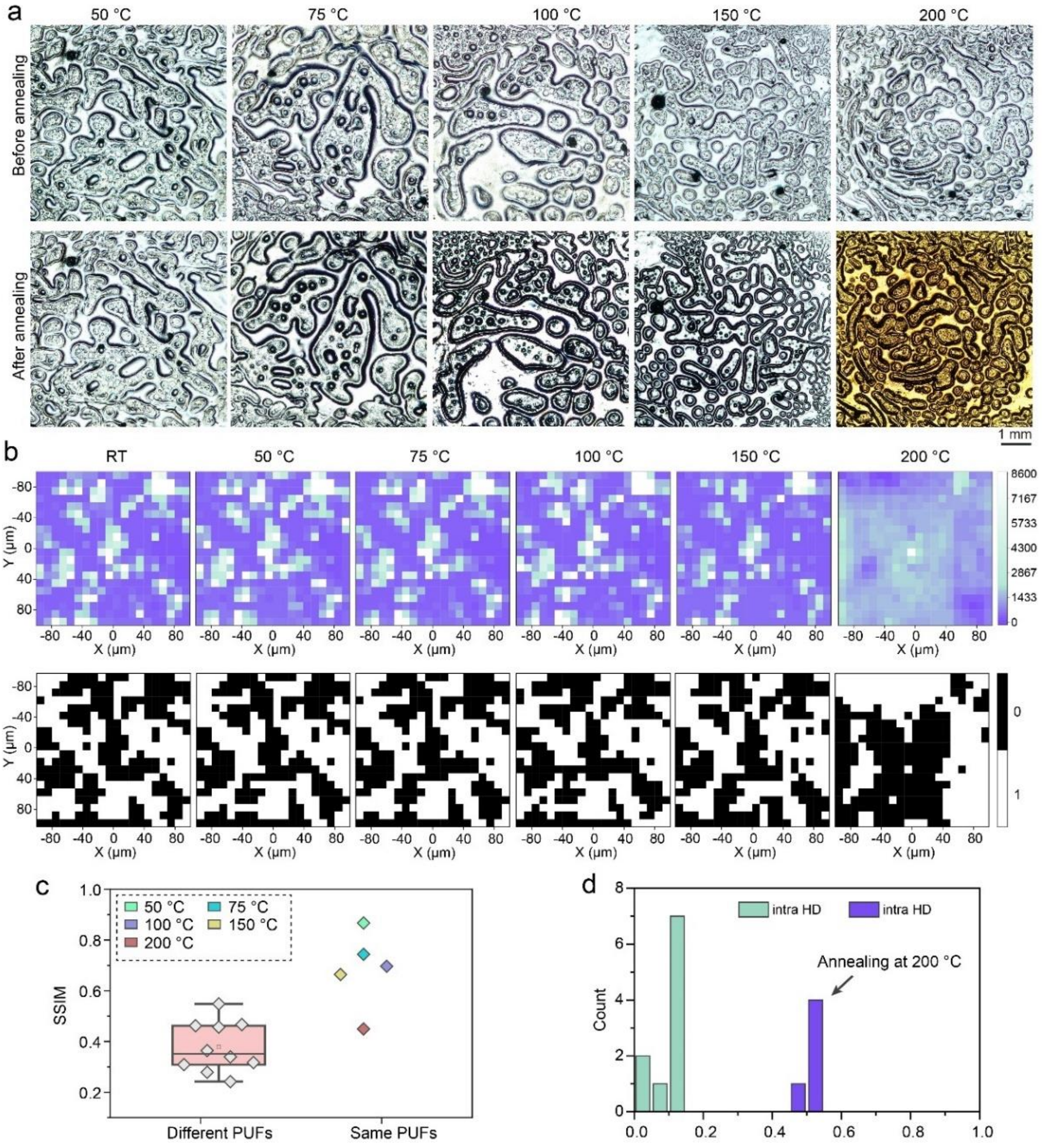

Figure S17. Readout and authentication of hierarchical PUF tags after annealing at different temperatures. (a) Representative examples of visual PUF labels processed at different temperatures. (b) PL mapping images and the corresponding binary encoding matrices of spectral PUF measured in situ at different temperatures. (c) Box plot of SSIM values extracted from visual PUF patterns processed at different temperatures. (d) Statistical distribution of intra-HDs of spectral PUFs measured at different temperatures.

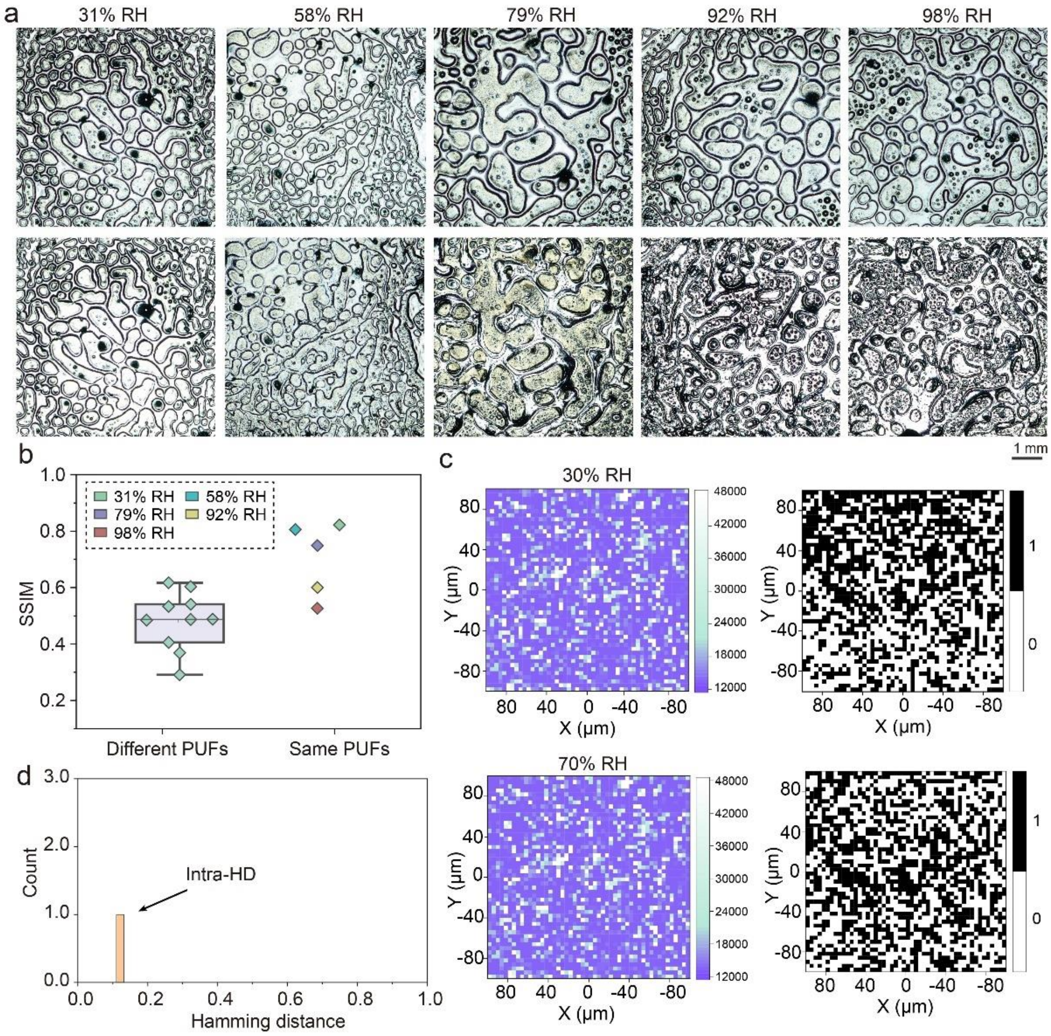

Figure S18. Readout and authentication of hierarchical PUF tags at different relative humidity (RH). (a) Representative examples of visual PUF labels processed at different RH. (b) Box plot of SSIM values extracted from visual PUF patterns processed at different RH. (c) PL mapping images and the corresponding binary encoding matrices of spectral PUF measured at different RH. (d) Statistical distribution of intra-HDs of spectral PUFs measured at different RH.

Table S1. Properties comparison of several multi-level PUFs

	Multiple physical features (Morphological, Optical, Electrical, Magnetic, etc.)	Various information carriers	Different scales	Multiple Authentication approach	Biocompatibility
Kim et al. [11]	✔	✘	✘	✘	—
Minh et al. [12]	✘	✘	✔	✔	✘
Zhang et al. [15a]	✔	✘	✘	✘	✔
Wang et al. [15d]	✔	✘	✔	✘	—
Jing et al. [15e]	✘	✘	✔	✘	—

Zhang et al. [17a]	✔	✘	✘	✔	—
Sun et al.[17b]	✔	✘	✔	✔	✔
This work	✔	✔	✔	✔	✔
